# Interferon-λ_4_ (*IFNL4*) Transcript Expression in Human Liver Tissue Samples

**DOI:** 10.1371/journal.pone.0084026

**Published:** 2013-12-20

**Authors:** Ahmad Amanzada, Waltraut Kopp, Ulrich Spengler, Giuliano Ramadori, Sabine Mihm

**Affiliations:** 1 Department of Gastroenterology and Endocrinology, University Medical Center Goettingen, Goettingen, Germany; 2 Department of General Internal Medicine, University Hospital Bonn, Bonn, Germany; University of Sydney, Australia

## Abstract

Eradication of hepatitis C virus (HCV) infection, both spontaneous and treatment-induced, is marked by the wildtype allele C of a single nucleotide polymorphism upstream of the *IL28B* gene, rs12979860. This favorable allele was recently described to be in linkage disequilibrium with the wildtype allele TT of a dinucleotide polymorphism, ss469415590, located within a new protein-coding gene. While the TT allele introduces a frame-shift and disrupts the open reading frame, only the variant allele, ΔG, creates a novel type III interferon (IFN) protein, IFN-λ_4_/IFNL4. Absence of IFNL4 is thus supposed to favor resolution of HCV infection. As to date *IFNL4* mRNA transcription has only been investigated in polyI:C-stimulated primary human hepatocytes and not yet in HCV infection *in vivo*, this study analyzed *IFNL4* mRNA expression in human liver biopsy specimens. Samples were obtained from patients with a broad panel of disorders including no liver disease, liver diseases of non-viral etiology, chronic hepatitis B and chronic hepatitis C. Hepatic *IFNL4* transcripts were detectable exclusively in a subgroup of chronic hepatitis C patients (24/45). Their amounts were positively related to liver HCV RNA copy numbers (p = 0.0023, r = 0.56) suggesting that the hepatic viral load influences *IFNL4* transcription irrespective of *IFNL4* governing genotype. Both, the *IFNL4* creating allele ΔG (p<0.0001) and actual *IFNL4* transcription (p = 0.0015) were found to be correlated to the activation of IFN stimulatory genes (ISGs). By contrast, *IFNL4* ss469415590 genotypes were not found to be related to *IFN-λ_2/3_/IL28* or *IFN-λ_1_/IL29* gene expression. In conclusion, this study is the first report on intrahepatic transcript levels of the recently discovered *IFNL4* gene. Data indicate that HCV infection in particular might activate *IFNL4* transcription in the liver. It provides a possible explanation as to why hepatitis C patients show ISG stimulation in their livers in the apparent absence of an induction of other IFN subtypes.

## Introduction

Since its discovery in 1957 as an entity which interferes with viral growth, a bundle of interferon (IFN) subtypes have been identified and some of them have been developed as drugs [1]–[3]. Chronic hepatitis C virus (HCV) infection has been treated with human recombinant IFN-α_2_ for over two decades [1], while human recombinant IFN-λ_1_/IL29/IFNL1 is presently being clinically evaluated as a further treatment option [4], [5]. The lack of induction of endogenous IFNs in the liver of chronic hepatitis C patients [6]–[9], or the proteolytic inactivation of signaling adaptor proteins in sensory pathways by HCV protease NS3/4A [10], add to the rationale of treating hepatitis C with IFN-based regimens.

Genetic polymorphisms within the region of the *IFN-λ* genes (e.g., *IL28B* rs12979860) have been identified to be associated with both spontaneous and treatment-induced elimination of HCV infection [11]–[14]. However, the underlying mechanism has remained elusive to date. Similarly, the transcriptional baseline activation of IFN stimulatory genes (ISGs) appeared to be linked to the outcomes of antiviral HCV treatment [15], [16]; patients with low pretreatment ISG expression were good responders to IFN-based therapy. Accordingly, the favorable rs12979860 C allele was found to be associated with low hepatic ISG expression [15], [17]–[20]. These were counter-intuitive and puzzling observations because high spontaneous ISG expression - usually a marker of strong IFN-α activity - apparently desensitizes to external IFN-α.

By applying an RNA-sequencing approach in polyI:C stimulated primary human hepatocytes, Prokunina-Olsson and coworkers uncovered of a novel *IFN-λ* gene, *IFN-λ_4_*/*IFNL4*, which is located in-between *IFN-*λ*_3_* and *IFN-*λ*_2_* [21]. It covers the site of rs12979860 within its intron 1 and harbors an additional polymorphism in exon 1, ss469415590, which is in linkage disequilibrium (LD) with rs12979860. The major TT allele of this dinucleotide polymorphism causes a frame-shift and disrupts the *IFNL4* ORF, while the minor ΔG allele enables IFNL4 protein expression. As the *IFNL4* creating allele ΔG is correlated with the unfavorable rs12979860 allele T, data suggest that the presence of IFNL4 might hamper eradication of HCV infection. Moreover, a transient overexpression of IFNL4 in hepatoma cells *in vitro* stimulated the expression of ISGs [21], which is consistent with the observation of a high baseline activation of ISGs in poor treatment responders.

By sequencing the putative regulatory region upstream of *IL28B*, this dinucleotide polymorphism was also described by Bibert and colleagues [22] and found to significantly better predict HCV clearance than rs12979860 [22], [23]. The authors showed the variant allele ΔG to create a methylation site in a CpG region and to control *IL28B* mRNA expression in polyI:C stimulated peripheral blood cells *in vitro* [22].

As *IFNL4* mRNA expression has not yet been elucidated in HCV infection *in vivo* to date, we analyzed *IFNL4* transcription in human liver samples with respect to the ss469415590 genotypes. Liver tissue samples were obtained from a broad range of patients with different disorders including minimal or no hepatic disease, liver diseases of non-viral etiology, chronic hepatitis B and chronic hepatitis C. In liver samples from patients with chronic hepatitis C, we additionally determined hepatic viral loads and *IL28A/B* and *IL29* mRNA expression and tested whether the relationship between *IFNL4* and ISG activation found *in vitro* is also existent *in vivo*.

## Patients and Methods

### Ethics Statement

This study was approved by the ethics committee of the University of Goettingen, Goettingen, Germany, on 13 July 2010 (chair Prof. Dr. Wolfgang Poser) and conformed to the current ethical principles of the Declaration of Helsinki. Informed written consent was obtained from each patient.

### Patients

Liver biopsy specimens were available from a total of 100 patients of Western European descent with chronic hepatitis C (n = 45; 21 males, 24 females; mean age 45.9±12.3 years), chronic HBV infection (n = 18; 14 males, four females; mean age 34.4±11.7 years), non-viral liver diseases (n = 25; 14 males, 11 females; mean age 45.5±12.6 years) and patients in whom liver disease could be ruled out by biopsy (n = 12; seven males, five females; mean age 51.8±14.9 years). Thus, the latter group can be referred to as ‘healthy liver tissues’. Tissues samples were snap frozen in liquid nitrogen and stored at −80°C until further use.

Diagnosis of chronic HCV infection was based on detection of HCV-specific antibodies and HCV RNA in serum for more than six months. Chronicity of liver disease was also confirmed by histological examination. Patients with other viral infections and patients with continued alcohol or drug abuse were excluded from this group. Diagnosis of chronic HBV infection was based on detection of HBs antigen, anti-HBc antibodies and HBV-DNA. Histopathological findings and diagnoses of patients with non-viral liver diseases comprised primary biliary cirrhosis (n = 2), primary sclerosing cholangitis (n = 2), autoimmune hepatitis (n = 1), hemochromatosis (n = 1), steatosis or steatohepatitis (n = 5), non-specific fibrosis (n = 2), ethanol-induced liver disease (n = 1) or cirrhosis (n = 1), cryptogenic liver cirrhosis (n = 1), non-Hodgkin lymphoma (n = 1) and hepatopathy (n = 8). Healthy liver tissue comprised samples taken from patients with slightly elevated serum transaminase activities (n = 4) and increased isolated γ-GT activities (n = 2), exclusion of focal nodular hyperplasia (n = 1) as well as baseline biopsies from donor livers assessed during the course of living liver donation (n = 5).

### Isolation of nucleic acids

Viral nucleic acids were isolated from serum samples (140 µl) by applying the spin protocol of the QIAamp Viral RNA Mini Kit (Qiagen) according to the supplier's instructions.

Genomic DNA and total cellular RNA were simultaneously isolated from liver biopsy specimens, which had been disrupted and homogenized by a rotor-stator homogenizer, by using the AllPrep DNA/RNA Mini Kit (Qiagen). RNA concentration and purity were checked in a photometer at λ = 260 nm and λ = 280 nm. RNA integrity was ascertained by 0.6% agarose gel electrophoresis.

### Determination of HCV genotype and hepatic viral loads

HCV genotyping was performed by using the Innolipa HCV II line probe assay (Innogenetics). HCV viral loads were assessed by 5′-nuclease assays using a primer pair from the 5′ UTR and a mixture of two FAM-labeled TAMRA TaqMan probes that recognize most of the viral subtypes with comparable amplification efficiency (forward 5′-CAG AAA GCG TCT AGC CAT GG-3′, reverse 5′-CGC AGA CCA CTA TGG CTC TC-3′, probe 1a/3 5′-TAG TAY GAG TGT CGT GCA GCC TCC AGG-3′, probe 1b 5′-TTA GTA TGA GTG TTG TGC AGC CTC CAG G-3′) [24]. Data were normalisedto the housekeeping gene *GAPDH* as a reference.

### Genotyping of IFNL genes

*IL28* rs12979860 genotyping was performed using a Custom TaqMan SNP Genotyping assay as described previously [25]. Genotyping of the dinucleotide polymorphism *IFNL4* ss469415590 (refSNP number rs368234815) was performed using the published primer and probes [21].

All reactions (10 µl, 4 ng genomic DNA) and analyses were carried out in the sequence detection system StepOne Plus (Applied Biosystems, Darmstadt, Germany). Ten µl reactions containing 5 ng genomic DNA was run in TaqMan Genotyping Master Mix (life technologies) according to the supplier's instructions.

### Quantification of hepatic gene expression

Total cellular RNA was reverse transcribed as described previously [7]. Complementary DNA (cDNA) corresponding to 6.4 ng of RNA was amplified using commercially available validated assays for *GAPDH* (Hs99999905_m1), myxovirus resistance protein A (*MxA*) (Hs00182073_m1), IFN-induced protein 44 (*IFI44*) (Hs00197427_m1) and TaqMan Universal Master Mix (life technologies) in 10 µl reactions.

For specific quantification of the *IFNL4* allelic transcript variants JN806234 (dG) and JN806227 (TT), primer and probes were designed to discriminate between the ten reported transcripts [21]. Forward and reverse primers were chosen complementary to sequences within exon 3 (5′-GAG GGA TGT GGC GGC CTG-3′) and exon 5 (5′-GAC CAC GCT GGC TTT GCG-3′), respectively, and a FAM-labeled minor groove binder (MGB) probe (5′-CCC GGA GAG CGG AC-3′) spanning the exon 4–5 boundary to further enhance signal specificity. A database search with BLASTN (NCBI) revealed complete and highly significant matches for both primers and probe only for the *IFNL4* RNAs (E = 0.007 for forward and reverse primers each, and E = 0.85 for the probe). The expected size of the amplicon of 129 bp was confirmed by electrophoretic separation using lab-on-a-chip technology in an Agilent 2100 bioanalyzer (DNA 1000 LabChip kit, Agilent Technologies, Boeblingen, Germany). The sequence of the amplicon was confirmed by automated custom sequencing (SeqLab Goettingen, Germany). Reaction efficiency was assessed to be 108.2%±12.7% (n = 5) by titrating samples and by using the integrated tool of the ABI software. Moreover, spiking of non-hepatitis C samples with hepatitis C samples with detectable amounts of *IFNL4* mRNA revealed complete recovery of the transcripts.

To rule out cross-contaminations, the following general precautions were taken: (i) non-template controls were carried along on each microtiter plate, (ii) RT-minus controls were conducted, and (iii) PCR reactions were exposed to UV irradiation before adding the cDNA sample and prior to amplification. To rule out a carryover specifically from samples from ΔG allele carriers to samples from TT homozygotes, we have applied the ss469415590 genotyping assay to cDNA preparations. In cDNA samples from heterozygotes, TT and ΔG transcripts were found in comparable amounts. cDNA preparations from TT homozygotes, in contrast, were found to contain only TT transcripts indicating that the *IFNL4* gene expression result does not derive from contaminating *IFNL4* ΔG transcripts.

Primer and probes for quantitation of *IL28A/B* and *IL29* gene expression were as follows: *IL28* reverse 5′-GGC ATC TTT GGC CCT CTT AAA-3′, forward 5′-GCC ACA TAG CCC AGT TCA AGT C-3′, probe FAM 5′-CTC CAC AGG AGC TGC-3′ MGB; *IL29* reverse 5′-AGG GTG GGT TGA CGT TCT CA-3′, forward 5′-CAC GCG AGA CCT CAA ATA TGT G-3′, probe FAM 5′-CCG ATG GGA ACC-3′ MGB. Please note that the assay for *IL28A/B* does not discriminate between *IL28A* and *IL28B*. Data were normalised to the amount of *GAPDH* transcripts. Reactions and analyses were carried out in the sequence detection system StepOnePlus according to the supplier's instructions.

### Statistical analysis

Univariate comparisons were calculated with the PC-STATISTIK software package version 4.0 (Hoffmann-Software Giessen, Germany). *P*-values of less than 0.05 were considered statistically significant.

Exact test for deviation from Hardy-Weinberg equilibrium was performed using the online calculator provided by the Institute of Human Genetics, Helmholtz Center Munich, Germany (http://www.ihg.gsf.de/cgi-bin/hw/hwa1.pl). Cubic exact solution web software CubeX (http://www.oege.org/software/cubex/) was used to determine LD (D′ and r^2^) [26].

## Results

### Cohort

The study cohort included patients with ‘healthy liver tissue’ after histological exclusion of liver disease (n = 12), patients with non-viral liver-diseases (n = 25), those with chronic HBV (n = 18) and patients with chronic HCV infection (n = 45). When considering all patients as one group (n = 100), distribution of *IL28* rs12979860 and *IFNL4* ss469415590 genotypes were in Hardy-Weinberg equilibrium (Table 1). As expected for a Western European population, the *IL28* rs12979860 wildtype allele C was closely correlated to the *IFNL4* ss469415590 wildtype allele TT as revealed by linkage disequilibrium analysis (D′ = 1, *r^2^* = 0.899).

**Table 1:** Patient genotype distribution.

	Genotype distribution			MAF	*P*
	wt/wt^a^	wt/v	v/v		
**IL28 rs12979860 [C/T]** a					
All (n = 100)	42 (42.0%)	47 (47.0%)	11 (11.0%)	0.345	0.6896^b^
Non-hepatitis C patients (n = 55)	31 (56.4%)	18 (32.7%)	6 (10.9%)	0.270	0.0035^c^
Hepatitis C patients (n = 45)	11 (24.4%)	29 (64.4%)	5 (11.1%)	0.433	
**IFNL4 ss469415590 [TT/ΔG]** a					
All (n = 100)	43 (43.0%)	45 (45.0%)	12 (12.0%)	0.345	0.9655^b^
Non-hepatitis C patients (n = 55)	31 (56.4%)	17 (30.9%)	7 (12.7%)	0.280	0.0052^c^
Hepatitis C patients (n = 45)	12 (26.7%)	28 (62.2%)	5 (11.1%)	0.422	

a [wildtype (wt)/variant (v)];

b Exact test for Hardy-Weinberg equilibrium;

c χ^2^-test for genotype distribution, non-hepatitis C patients *vs* hepatitis C patients;

MAF: Minor allele frequency.

Comparing hepatitis C with non-hepatitis C patients, rs12979860 as well as *IFNL4* ss469415590 genotype distributions were significantly different with a higher minor allele frequency among hepatitis C patients (Table 1). This shift in genotype distribution towards the minor allele is consistent with the concept that the wildtype allele was protective in our patients with hepatitis C. Hepatitis C TT homozygotes did not differ significantly from carriers of the ΔG allele regarding distribution of HCV types, age, serum transaminase activities or histological manifestations (Table 2).

**Table 2:** Patient characteristics.

	All	IFNL4 ss469415590 genotype			p-value
		TT/TT	TT/ΔG	ΔG/ΔG	
**Healthy livers**	12	7	5	0	
**Non-viral liver diseases**	25	12	7	6	
**Chronic hepatitis B**	18	12	5	1	
**Chronic hepatitis C**	45	12	28	5	
HCV types 1a/1b/3 [n]	7/29/5	2/7/3	5/18/2	0/4/0	0.4030
age [mean ± SD]	45.9±12.3	41.3±11.2	48.1±12.3	44.0±14.3	0.2657
serum transaminase activity					
ALT [U/l]	76±61	82±45	78±71	52±36	0.6403
AST [U/l]	45±37	54±59	45±33	34±20	0.6422
inflammatory activity					
mild	21	5	13	3	0.4957
moderate/severe	19	7	11	1	
fibrosis					
absent/mild	26	7	17	2	0.6099
moderate/severe/cirrhosis	14	5	7	2	
steatosis					
absent	18	8	7	3	0.0459
mild/moderate/severe	22	4	17	1	

### *IFNL4* transcript expression in human liver samples

**Figure 1:**
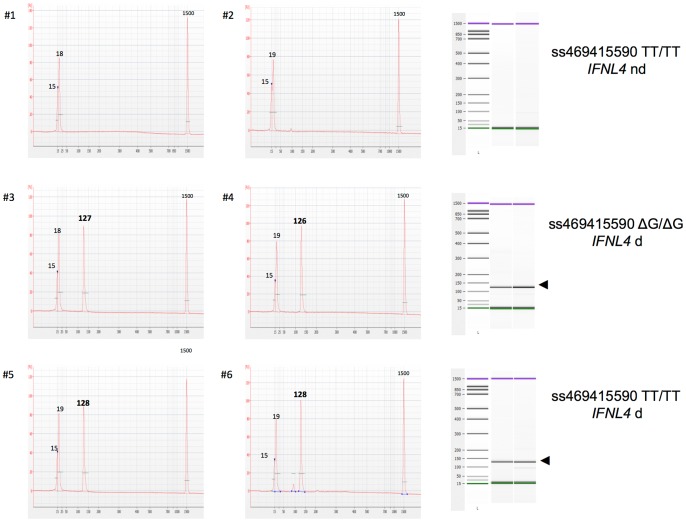
Electropherograms of *IFNL4* amplicons. After completion of PCR amplification, 1 µl of samples was separated and analyzed in the Agilent 2100 bioanalyzer using the DNA 1000 LabChip kit. Electropherograms and gels are shown for two representative samples each of ss469415590 TT homozygotes without any amplification of *IFNL4*-specific transcripts (*IFNL4* nd) (#1, #2) or of ΔG homozygotes (#3, #4) and of TT homozygotes (#5, #6) with detectable amounts of hepatic *IFNL4*-specific mRNA (*IFNL4* d). Analysis confirmed the lack of products in samples #1 and #2, and the presence of products of the expected size (accuracy ±5 bp, as given by the supplier) both in ΔG and TT homozygotes.

Among the ten novel transcripts identified by Prokunina-Olsson and colleagues in polyI:C stimulated primary human hepatocyte cultures upstream of *IFNL3*, only p179 (NM_001276254) was designated as an IFN-λ protein, *IFNL4*, on the basis of a 29% protein sequence homology with type III IFNs [21]. In order to specifically quantify *IFNL4* mRNAs in liver specimens, an assay was designed as outlined in the Patients and Methods section. Specificity of the assay was validated as follows: (i) a BLASTN search revealed a lack of homologies of the primers or probe to any other genes, in particular of *IFN-λs*, (ii) the expected size of the amplicon (129 bp), or the absence of any or any further products, respectively, was confirmed by high resolution electrophoretic separation (Figure 1) and (iii) custom sequencing confirmed correspondence of the amplicon with the reference sequence (four representative samples, two TT and ΔG homozygotes each, data not shown) (see Patients and Methods section).

**Figure 2:**
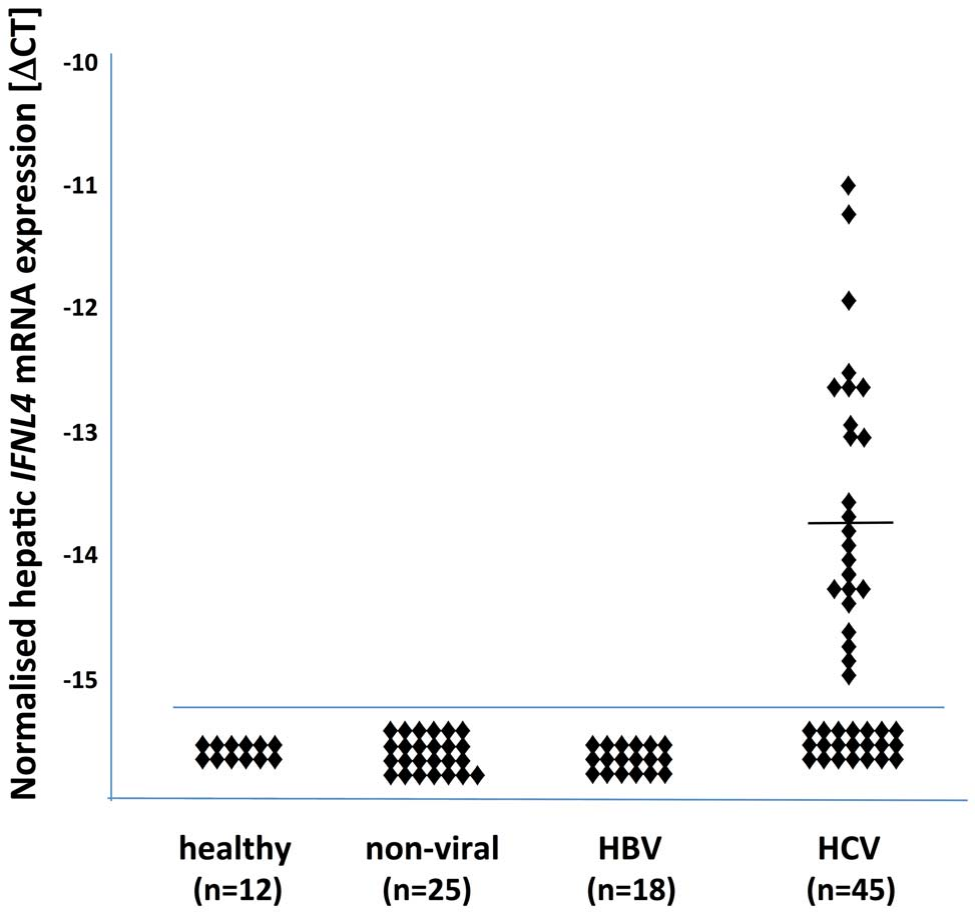
*IFNL4* transcript expression in human liver samples. Liver biopsy specimens taken from patients with suspected but later excluded liver diseases (healthy controls) and from patients with non-viral liver diseases, chronic HBV infection or chronic HCV infection were analyzed for *IFNL4* gene expression. *IFNL4* gene expression was quantified by applying a 5′-nuclease assay using a specific primer-probe set as outlined in the Patients and Methods section. Data were normalised to *GAPDH* transcripts as a reference.

In the various patient groups in this study, *IFNL4* transcripts were exclusively detected in liver specimens from patients with hepatitis C, in fact in 24/45 (53.3%) of the samples (Figure 2). Non-detectability of *IFNL4* transcripts due to insufficient amounts of cDNA could be ruled out as we matched both groups for the number of reference GAPDH transcripts. With a ratio of *IFNL4/GAPDH* transcripts of 1/12,416 on average (range 1/2,194 to 1/30,573, lower limit of detection) in whole liver tissue samples, *IFNL4* mRNA expression was markedly lower than that of *IL28A/B* or *IL29* (4.3-fold and 5.6-fold, respectively, data not shown).

**Figure 3:**
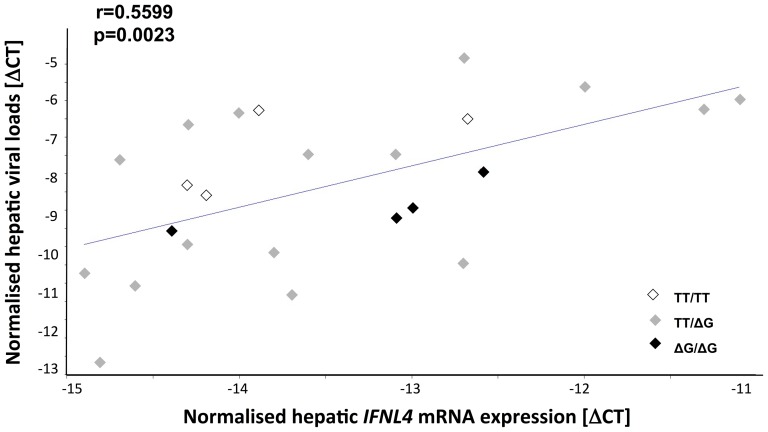
The number of hepatic *IFNL4* transcripts relates to hepatic viral loads. A linear regression analysis among hepatitis C patients with measurable amounts of *IFNL4* mRNA revealed a correlation between hepatic viral loads, which was assessed by the amount of viral nucleic acids in relation to the amount of *GAPDH* transcripts, and hepatic *IFNL4* transcripts, which were also normalised to the amount of *GAPDH* transcripts. Correlation coefficient and level of significance are given. Symbols indicate patient genotypes.

*IFNL4* transcripts were detectable in patients of all three genotypes, yet not in all of the ΔG allele carriers (distribution of genotypes (TT/TT∶TT/ΔG∶ΔG/ΔG)- was 4∶16∶4 and 8∶12∶1 in patients with detectable and non-detectable quantities of *IFNL4* transcripts, respectively, p = 0.1720). The amount of *IFNL4* steady state mRNA levels was not found to differ with regard to ss469415590 genotypes (p = 0.8125, data not shown). However, it was found to be closely related to intrahepatic HCV viral loads (r = 0.5599, p = 0.0023) (Figure 3). As the group of patients with detectable amounts of *IFNL4* transcripts comprises individuals with all three genotypes (TT/TT, TT/ΔG, ΔG/ΔG), among them the wildtypes which are not supposed to generate any protein, and the heterozygotes and rare allele homozygotes supposed to express different amounts of protein, data are indicative for a relationship between hepatic viral loads and *IFNL4* transcriptional (promoter) activity irrespective of the *IFNL4* ORF governing ss469415590 genotype (see symbols in Figure 3).

### Hepatic activation of ISGs and *IL28A/B* and *IL29* transcription

**Figure 4:**
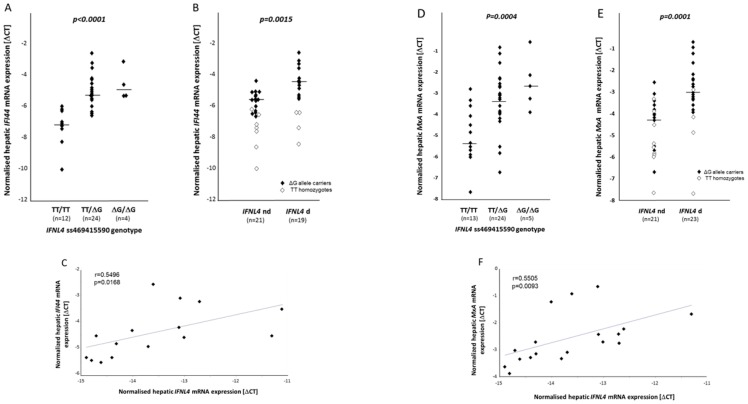
Hepatic activation of ISGs. Liver samples from chronic hepatitis C patients were quantified for *IFI44* (A, B) and *MxA* (D, E) transcripts with regard to *IFNL4* ss469415590 genotypes and detectability of hepatic *IFNL4* transcripts. Medians are indicated by horizontal bars. Carriers of the ΔG allele experienced significantly higher ISG expression than TT homozygotes (A, D), as do ΔG allele carriers (closed symbols) with detectable amounts of hepatic *IFNL4* transcripts (*IFNL4* d) when compared to those without (*IFNL4* nd) (B, E). Open symbols indicate TT homozygotes, data of whom were not included in significance testing. Linear regression analyses revealed positive relationships between *IFNL4* gene transcripts and *IFI44* (C) and *MxA* (F) mRNA expression.

The rs12979860 unfavorable minor allele T, which marks the *IFNL4* creating ss469415590 allele ΔG, has been shown to be associated with higher basal hepatic ISG activation. Furthermore, transient expression of *IFNL4* was reported to activate expression of ISGs in hepatoma cells *in vitro* [21]. We thus related the *IFNL4* ss469415590 genotypes to ISG activation in human liver samples. *IFI44 (p44)* was chosen since p44 was the first cytoplasmic protein formerly shown to be associated with a characteristic ultrastructural entity (membranous web) within hepatocytes in nonA-nonB hepatitis, and it was later on found to be IFN-inducible [6]. *MxA*, one of the first ISGs described, is among those reported by Prokunina-Olsson et al. to be activated by IFNL4 [21]. Carriers of the *IFNL4* creating ΔG allele were found to have significantly higher amounts of *IFI44* mRNA (Figure 4A) or *MxA* mRNA (Figure 4D) than patients homozygous for the disruptive TT allele. Moreover, by relating actual *IFNL4* transcription in carriers of the ΔG allele to ISG induction, patients with measurable quantities of *IFNL4* mRNAs presented significantly stronger ISG induction than those without (Figure 4B, E). Finally, linear regression analyses revealed positive correlations between both I*FI44* and *MxA* transcripts and *IFNL4* gene transcripts in ΔG allele carriers (Figure 4C, F). These findings are in line with the concept that IFNL4 activates ISGs [21].

In view of the high LD between rs12979860 and ss469415590, and since many reports described an absence of relationship between rs12979860 genotypes and hepatic *IFN-λ* gene expression [17]–[19], [27], expectedly, neither hepatic *IL28A/B* nor *IL29* mRNA expression were found to be related to ss469415590 genotypes (p = 0.8820 (n = 43) and p = 0.4115 (n = 41) for *IL28A/B* and *IL29*, respectively, data not shown).

## Discussion

Here we report on a first cross-sectional analysis of *IFNL4* gene transcripts in liver biopsies from patients with hepatitis C, hepatitis B, various inflammatory liver diseases due to other etiologies, and from patients without any histological liver disease. In this broad range of samples, *IFNL4* mRNA transcripts were exclusively detected in specimens from patients with chronic hepatitis C, albeit only in half of them. This finding suggests that the *IFNL4* gene is specifically activated in HCV infection. Evidence for this is further strengthened by detection of a quantitative correlation between HCV viral loads and the amount of *IFNL4* transcripts. Thus, HCV infection possibly induces hepatic *IFNL4* gene transcription. This is in line with the finding that polyI:C stimulates *IFNL4* activation in primary human hepatocytes *in vitro* [21].

In contrast to the *IFNL4* allelic ΔG transcript variant (JN806234), the allelic TT transcript (JN806227) is, once transcribed, suggested to most likely be eliminated by nonsense-mediated RNA decay due to a premature stop codon [21]. As we found comparable amounts of transcripts in TT and ΔG homozygotes (n = 4 each), we have no evidence for a difference in stability of these two transcript variants in freshly *ex vivo* derived liver biopsy specimens.

Regarding the IFNL4 protein, we have no information. As only the ΔG transcript variant is IFNL4 protein encoding, the amount of transcripts cannot be taken as a measure of gene expression. This is true particularly for carriers of the TT allele. However, the amount of transcripts should reflect, with reservations, transcriptional (promoter) activity.

As for non-measurable quantities of *IFNL4* transcripts in liver samples we can only speculate. This finding possibly resembles a low abundance of *IFNL4* transcripts below the limit of detection. However, the positive relationship between *IFNL4* transcript expression and hepatic viral loads over its entire range and our matching for the reference *GAPDH* argue against this possibility. It could be related to a differential sensing of HCV RNA *via* intracellular and extracellular/endosomal pattern recognition receptors in liver cells [28] or to efficient cleavage of adaptor molecules in only some of the samples. The *in vitro* experiments reported by Prokunina-Olsson *et al.* studied primary human hepatocyte cultures stimulated with polyI:C, where absence of the HCV NS3/4A protease could have allowed an unhindered activation of *IFNL4* [21].

We and others showed that HCV infection in humans is not accompanied by an activation of type I or type III IFNs in the liver [7]–[9], [29], [30]. These results appear to be partly due to the proteolytic cleavage of the adaptor molecules of the cytoplasmatic and the endosomal driven sensory pathways by HCV NS3/4A protease in the virus-infected compartment of liver cells [10]. In this regard, induction of *IFNL4* mRNA in hepatitis C liver tissue is surprising as it is apparently the only IFN in the liver shown to date to be clearly inducible by HCV infection in humans. It appears that *IFNL4* induction is less or not at all affected by an inactivation of signaling molecules as seen in the other IFNs.

With regard to genotype, carriers of the *IFNL4* creating ΔG allele experienced higher ISG activation than *IFNL4* disrupting TT homozygotes. To our current knowledge, this finding might be attributable to the ability to express *IFNL4* [21] or to a decreased *IL28B* expression [22]. Our data clearly show that the first possibility applies since the effective *IFNL4* transcriptional activity was found to be related to ISG stimulation as well. Regarding the second possibility, we cannot draw any conclusion as our assay does not discriminate between the *IL28* paralogues *A* and *B*, and because of the high LD of the two loci and only few genotype discordant samples.

In conclusion, our data, obtained from a relatively broad panel of human liver tissue samples of various etiology, demonstrate exclusive induction of *IFNL4* mRNA in livers of chronic hepatitis C patients, albeit only in a subgroup of them. This is remarkable in view of an otherwise attenuated hepatic *IFN* gene activation in HCV infection in humans. An obvious associative evidence of the ΔG allele with the activation of ISGs could be attributed to liver *IFNL4* mRNA expression in our study patients.
